# Bloodstream infections in pediatric hematology/oncology patients: Six years’ experience of a single center in Turkey

**DOI:** 10.3906/sag-1812-101

**Published:** 2019-08-08

**Authors:** Tuğçe TURAL KARA, Tuğba ERAT, Aysun YAHŞİ, Halil ÖZDEMİR, Talia İLERİ, Elif İNCE, Nurdan TAÇYILDIZ, Emel ÜNAL, Ergin ÇİFTÇİ, Erdal İNCE

**Affiliations:** 1 Department of Pediatric Infectious Diseases, Ankara University Faculty of Medicine, Ankara Turkey; 2 Department of Pediatric Hematology, Ankara University Faculty of Medicine, Ankara Turkey; 3 Department of Pediatric Oncology, Ankara University Faculty of Medicine, Ankara Turkey

**Keywords:** Bloodstream infections, hematology and oncology, microorganisms

## Abstract

**Background/aim:**

Bloodstream infections are the major cause of morbidity, increased cost, prolonged hospitalization, and mortality in pediatric patients. Identifying the predominant microorganisms and antimicrobial susceptibilities in centers helps to select effective empirical antimicrobials which leads to positive clinical outcomes. We aimed to identify the causative microorganisms and their antimicrobial susceptibilities in patients with bloodstream infections.

**Materials and methods:**

Data belonging to patients with hematological and/or oncological diseases admitted to our hospital with fever between January 2010 and November 2015 were analyzed.

**Results:**

In total, 71 patients who had 111 bloodstream infection episodes were included. Responsible pathogens were detected as follows: 35.1% gram-positive microorganisms, 60.5% gram-negative bacteria, and 4.4% fungi. The most common causative gram-negative pathogen was *Escherichia coli* and the most commonly isolated gram-positive microorganism was coagulase-negative staphylococci.

**Conclusion:**

Gram-negative microorganisms were predominant pathogens in bloodstream infections.* Escherichia coli* and coagulase-negative staphylococci were the most commonly isolated responsible pathogens. Beta-lactam/lactamase inhibitors were suitable for empirical treatment. However, in critical cases, colistin could have been used for empirical treatment until the culture results were available. Routine glycopeptide use was not required. By identifying the causative microorganisms and their antimicrobial resistance patterns, it will be possible to obtain positive clinical results.

## 1. Introduction

Bloodstream infections (BSIs) are the main cause of morbidity, prolonged hospitalization, increased cost, and mortality in patients [1,2]. Underlying diseases, applied chemotherapy protocols, central venous catheter (CVC), radiation therapy, surgical procedures, and mucositis may predispose patients to infections [3]. Today, more than 80% of pediatric patients receiving cancer treatment have long-term CVCs [4]. The majority of BSIs are related to the use of these catheters in children. 

Early diagnosis and empirical antibiotic treatment may help improve the prognosis of BSIs. However, the choice of empirical antibiotic treatment must be based on the patient’s clinical status, frequently detected isolates, and their antimicrobial susceptibility patterns in the region [5]. In some hospitals, treatment of BSIs caused by multiresistant bacteria is an important problem because of increasing antibiotic resistance [6].

Until the 1990s gram-positive microorganisms were the pathogens most often responsible for BSIs; today gram-negative pathogens are frequently isolated in many centers [7]. It is very important to know the local prevalence of responsible microorganisms for appropriate management. For this purpose, in our study we aimed to identify the microorganisms that were isolated from blood culture during the febrile period in pediatric hematology and oncology patients, their antimicrobial susceptibility patterns, and clinical outcomes.

## 2. Materials and methods

### 2.1. Study populations

This was a cross-sectional study that included febrile patients with hematological and/or oncological diseases that were diagnosed as BSIs, followed between January 2010 and November 2015. Demographic and clinical characteristics of patients were collected from the database retrospectively. Approval for the study was obtained from the Ethics Committee of Ankara University Faculty of Medicine (2016/04).

### 2.2. Case definitions 

Patients met the following criteria: (1) hematological and/or oncological disease diagnosis, (2) fever (body temperature ≥38 C° once or ≥37.5 C° for at least 2 h, measured with an infrared tympanic thermometer), and (3) positive blood culture result (BSI or CRBSI). Neutropenia was defined as an ANC <1000 cells/µL, with severe neutropenia as ANC <500 cells/µL. If CRP level was <5 mg/L, it was accepted as negative. 

Bloodstream infection (BSI) was defined as 1 positive blood culture with pathogenic microorganisms or ≥2 separately positive blood cultures with nonpathogenic microorganisms of normal skin flora (CNS (coagulase-negative staphylococci), *Streptococcus viridans*, *Propionibacterium* spp., *Bacillus *spp.) that were not related to an infection at another site.

Diagnosis of catheter-related bloodstream infection (CRBSI) was based on the Infectious Disease Society of America (IDSA) clinical and practice guidelines for the diagnosis and management of intravascular catheter related infections—2009 update, which contains the following criteria: detecting the same microorganism on the catheter and peripheral blood culture, and microorganism’s reproduction time is detected at least 2 h earlier in catheter blood culture than in peripheral blood culture [8]. These pathogens were not related to an infection at another site. 

Clinical signs of infection (fever, chills, and hypotension) were detected at the same time as positive blood culture. Asymptomatic bacteremia was not considered an infection. 

The exclusion criteria were as follows: (1) antibiotic treatment prior to admission to the hospital, (2) absence of simultaneous catheter and peripheral blood culture, (3) asymptomatic patients, (4) one positive blood culture with nonpathogenic microorganisms of normal skin flora.

### 2.3. Culture and identification

During the febrile period, blood samples of at least 2 mL were taken simultaneously from the peripheral vein and both of the catheter lumens. All blood samples were taken after the application of septic processes and cultured with a BACTEC 9240 system (Becton Dickson, Sparks, MD, USA). Antimicrobial susceptibility patterns of microorganisms were detected with the Phoenix 100 ID/AST system (Becton Dickinson, Phoenix 100, MD, USA) according to the Clinical and Laboratory Standards Institute (CLSI) guidelines. 

### 2.4. Treatment

All patients with BSIs were treated with systemic antimicrobial drugs. According to the Infectious Disease Society of America (IDSA) guidelines, for empirical treatment, antipseudomonal broad-spectrum beta-lactamase antibiotics were used if ANC <500 /mm3, or third generation cephalosporin was used if ANC >500 /mm3 and there were no signs of sepsis [8]. In catheter-related bloodstream infections (CRBSIs), catheter removal was performed under the following conditions: *S. aureus*,* Pseudomonas* spp., and fungus related catheter infections, nontunneled catheter infections, all tunnel infections, complicated catheter infections, and in septic patients. 

If catheter removal was not required, ALT was used with systemic intravenous antibiotics in patients documented with CRBSI, which includes concentrated antibiotic solution (vancomycin 5 mg/mL, gentamicin 1 mg/mL, amikacin 5 mg/mL, ciprofloxacin 0.2 mg/mL, ampicillin 10 mg/mL, ceftazidime 0.5 mg/mL, cefazolin 5 mg/mL), sterile normal saline, and heparin (100 U/mL). This mixture was introduced into the catheter lumen (usually 3 mL) and then the catheter was locked. Antibiotic lock solution was replaced every 24 h. The duration of ALT was varied according to the causative microorganisms; ALT treatment for *Enteroccoccus* spp. was 7–14 days, CNS was 10–14 days, and gram-negative bacilli was 10–14 days. Control catheter and peripheral blood cultures were performed after 72 h of treatment. Patients were followed up according to clinical signs. If fever was persistent and sterile blood culture could not be achieved, catheter was removed, and catheter tip culture was performed.

In addition, only systemic antibiotics were applied for BSIs in accordance with antimicrobial susceptibility. Patients with gram-negative microorganisms were usually treated for 14 days and gram-positive BSIs were commonly treated for 10 days. 

All patients were followed for at least 6 months.

### 2.5. Statistical analysis

SPSS version 18.0 (Chicago, IL, USA) was used for analysis of results. Categorical variables were compared using the chi-square test or Fisher’s exact test. Continuous variables were compared using the**t-test or Mann–Whitney U test. A P-value was considered statistically significant if it was detected as ≤0.05.

## 3. Results

Between January 2010 and November 2015, 71 pediatric patients with 111 BSIs were retrospectively included in the study. The median age of patients was 90 (range: 3–247) months. Male/female ratio was 1.7/1. Common underlying diseases were acute lymphoblastic leukemia (ALL) (33.8%), acute myeloid leukemia (AML) (28.2%), solid organ tumors (22.5%), aplastic anemia (4.2%), thalassemia major (4.2%), lymphoma (2.8%), myelodysplastic syndrome (1.4%), hemophagocytic lymphohistiocytosis (1.4%), and chronic myeloid leukemia (1.4%). Of 71 patients, 48 (67.6%) had 1 episode, 12 (16.9%) had 2 episodes, and 11 (15.5%) had ≥3 episodes. CRBSIs were identified in 80 (72.1%) episodes that included 58 Hickman catheters, 18 totally implantable ports, and 4 nontunneled central venous catheters. 

Eighty-nine (80.2%) episodes occurred during the neutropenic period; 83 (74.8%) of them had severe neutropenia. The median value of ANC was 100 (range: 0–16,800) cell/mm3. The mean level of CRP was 86.1 ± 79.1 (median: 65.1; range: 1–389) mg/L. The median duration of neutropenia was 10 (range: 1–525) days. Demographic and clinical features of patients are summarized in Table 1.

**Table 1:** Demographic and clinical features of patients with bloodstream infections.

Characteristic	n	Patients’ underlying disease	n (%)
Total number of patients	71	Acute lymphoblastic leukemia	24 (33.8)
Age, median (range), months	90 (3–247)	Acute myeloid leukemia	20 (28.2)
Male n (%)	45 (63.4)	Solid organ tumors	16 (22.5)
Total blood culture positivity	111	Aplastic anemia	3 (4.2)
Catheter related bloodstream infections	80	Thalassemia major	3 (4.2)
Catheter type		Lymphoma	2 (2.8)
Hickman	58	Myelodysplastic syndrome	1 (1.4)
Port	18	Hemophagocytic lymphohistiocytosis	1 (1.4)
Nontunneled central venous catheter	4	Chronic myeloid leukemia	1 (1.4)
C-reactive protein levels, mg/L	86.1 ± 79.1		
White blood count, cell/mm3	1907.2 ± 3471.9		
Absolute neutrophil count (ANC), cell/mm3	1187.4 ± 2985.3		
Number of episodes (ANC <1000 cell/mm3)	89		
Number of episodes (ANC <500 cell/mm3)	83		
Neutropenia duration (day)	23.1 ± 56.6		
Mortality	3 (2.7%)		

For a total of 111 BSIs, gram-positive microorganisms were responsible for 35.1%, gram-negative microorganisms were responsible for 60.5%, and 4.4% were due to fungi.* Escherichia coli* was the most commonly detected gram-negative pathogen (16.7%) and CNS was the most commonly isolated gram-positive microorganism (16.7%). Common responsible microorganisms detected in CRBSIs were as follows: CNS (18.1%), *E. coli* (15.7%), *K. pneumoniae *(13.3%),* Pseudomonas *spp. (12.0%), alpha-hemolytic *Streptococcus *(7.2%), *Enterococcus *spp. (6.0%), *Candida *spp. (6.0%), *Acinetobacter* spp. (4.8%), and *Serratia marcescens *(4.8%). However, the most commonly identified pathogens in other BSIs were *E. coli* (19.6%), CNS (12.9%), *K. pneumoniae *(12.9%),* Enterococcus *spp. (12.9%), *S. aureus *(9.7%), and *S. maltophilia *(9.7%). Between 2010 and 2015, gram-negative microorganisms were more frequently causative for BSIs, though gram-positive pathogens were predominant in 2011. The distribution of causative microorganisms by year is given in Figure. Polymicrobial etiology was detected in 3 (2.7%) episodes. The causative microorganisms are given in Table 2.

**Figure:**
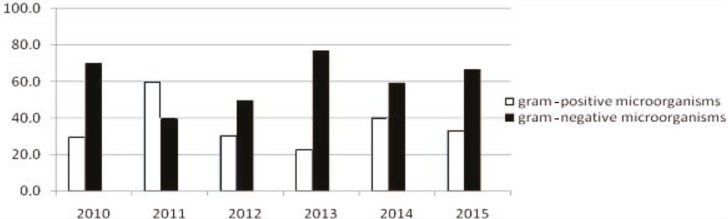
Distribution of causative microorganisms of bloodstream infections in pediatric patients by year.

**Table 2:** The responsible microorganisms of bloodstream infections.

	CRBSIsn (%)	Other BSIsn (%)	Total numbern (%)
E. coli	13 (15.7)	6 (19.6)	19 (16.7)
CNS	15 (18.1)	4 (12.9)	19 (16.7)
K. pneumoniae	11 (13.3)	4 (12.9)	15 (13.2)
Pseudomonas spp.	10 (12.0)	2 (6.5)	12 (10.5)
Enterococcus spp.	5 (6.0)	4 (12.9)	9 (7.9)
Acinetobacter spp.	4 (4.8)	2 (6.5)	6 (5.3)
Alpha-hemolytic Streptococcus	6 (7.2)	0 (0)	6 (5.3)
Serratia marcescens	4 (4.8)	1 (3.2)	5 (4.4)
S. aureus	2 (2.4)	3 (9.7)	5 (4.4)
Candida spp.	5 (6.0)	0 (0)	5 (4.4)
S. maltophilia	1 (1.2)	3 (9.7)	4 (3.5)
Enterobacter spp.	3 (3.6)	0 (0)	3 (2.6)
A. xylosoxidans	2 (2.4)	1 (3.2)	3 (2.6)
Orchobactrum anthropi	0 (0)	1 (3.2)	1 (0.9)
Leuconostoc spp.	1 (1.2)	0 (0)	1 (0.9)
Campylobacter jejuni	1 (1.2)	0 (0)	1 (0.9)
Total number	83 (100)	31 (100)	114 (100)

CNS: coagulase-negative staphylococci

In our study, unlike other gram-negative bacteria, *K. pneumoniae* had lower beta-lactam susceptibility. Extended spectrum beta-lactamase (ESBL) was produced by 60% of *Klebsiella *spp. and 42.1% of *E. coli.* In addition, 15.8% of *E. coli* and 26.7% of *Klebsiella *spp. produced carbapenemase. Colistin resistance was detected for only one gram-negative bacteria, *Acinetobacter *spp., and quinolone resistance of *E. coli *and *K. pneumoniae* was detected in 75% and 55.6%, respectively. *Enterococcus* spp. had 11.1% (1/9) ampicillin sensitivity and 33.3% (3/9) vancomycin resistance. Enterobacteriaceae had 16.2% carbapenem resistance. In addition, methicillin-resistant *Staphylococcus aureus* was not detected in our study. Antibiotic susceptibility patterns of causative microorganisms are given in Tables 3 and 4. 

**Table 3:** Gram-negative pathogens and their antibiotic susceptibility patterns (%).

	n	Ceftriaxone	Amikacin	Gentamicin	PT	CS	Meropenem	Ciprofloxacin	Colistin
E. coli	19	42.1	52.6	47.4	52.6	52.6	78.9	26.3	100
K. pneumoniae	15	33.3	86.7	66.7	53.3	53.3	86.7	53.3	100
Pseudomonas spp.	12	50	66.7	66.7	83.3	66.7	50	75	100
Acinetobacter spp.	6	0	33.3	33.3	50	50	60	50	100
S. marcescens	5	-	80	20	0	20	60	40	0
Enterobacter spp.	3	66.7	100	100	100	100	100	100	100
S. maltophilia	4	-	-	-	-	-	-	100	-
A. xylosoxidans	3	-	0	0	100	100	100	100	0

CS: cefoperazone/sulbactam; PT: piperacillin/tazobactam

**Table 4:** Gram-positive pathogens and their antibiotic susceptibility patterns (%).

	n	Ampicillin	SAM	Teicoplanin	Vancomycin	Linezolid
CNS	19	0	0	100	100	100
Enterococcus faecalis	1	100	-	100	100	100
Enterococcus faecium	8	0	-	37.5	37.5	100
Alpha-hemolytic Streptococcus	6	0	-	100	100	100
S. aureus	5	0	100	-	100	100

CNS: coagulase-negative staphylococci; SAM: sulbactam ampicillin

All patients with BSIs were treated with intravenous antibiotic therapy. The most commonly used antimicrobial agents were as follows: piperacillin tazobactam, cefoperazone sulbactam, meropenem, and ceftriaxone against gram-negative microorganisms, and teicoplanin and vancomycin against gram-positive pathogens. The mean duration of treatment was 11.3 ± 5.86 days.

Of these 111 episodes, 108 were successfully treated, but 7-day mortality occurred in 2.7% of episodes due to septicemia. Microorganisms that were responsible for mortality were as follows: *E. coli *(n = 1), *K. pneumonia *(n = 1), and *Serratia marcescens *(n = 1). The mean CRP level was 84.95 ± 79.69 (median, 63; range, 1–389) in the mortality group and 153.33 ± 54.90 (median, 161; range, 95–204) in surviving patients. Although there were no significant differences in white blood cells count, neutrophil count, duration of neutropenia, and catheterization duration, the CRP levels were significantly higher in the episodes that concluded with 7-days mortality (P < 0.001).

## 4. Discussion

Children with hematological and/or oncological disease have a high risk for infections due to neutropenia, immunosuppressive therapy, chemotherapy, radiotherapy, impaired mucosal barrier, and CVC [9]. Neutropenia is the most important predisposing factor [10]. In this study, 80.2% of episodes occurred in the neutropenic period and 77.8% of them had severe neutropenia. In the study by Kuo et al. neutropenia was identified in 83.5% of BSI episodes; 73% of them had severe neutropenia [11].

Responsible pathogens may vary between centers according to the use of empirical intravenous antibiotics and the rate of compliance with antisepsis procedures. Gram-positive microorganisms are reported as the most commonly responsible etiologic agents in many centers. This has been suggested as being linked to the following reasons: (1) microorganisms may be transmitted from colonized skin during the insertion of CVC; (2) deterioration of mucosal integrity with chemotherapeutic and radiotherapeutic treatment; and (3) antibiotic prophylaxis [12,13]. Some studies suggested that early generation quinolones may help to decrease the frequency of gram-negative infections. However, similar effect was not detected for gram-positive infections [14]. In the literature, gram-positive microorganisms were detected as being predominant in BSIs [9,15]. However, gram-negative bacteria were reported more often in some studies [11,16,17]. They suggested that gram-negative pathogens were three times more frequent than gram-positive organisms due to the low rate of catheter use and not being administered quinolone prophylaxis [16,17]. Ye et al. analyzed data belonging to children with acute leukemia; gram-negative bacteria (53.8%), gram-positive bacteria (45%), and fungi (1.2%) were isolated in BSIs. They suggested that semipermanent catheters that were inserted in all patients with acute leukemia were responsible for gram-positive bacteria causing BSIs. Therefore, they reported that the most frequently isolated pathogen was CNS, unlike other studies [18]. 

In our study, all patients had long and short-term central venous catheters. Although gram-negative organisms were detected at significantly higher rates, CNS (18.1%) was the most causative pathogen for CRBSIs. Celebi et al. reported similar results in Turkey. They analyzed data belonging to 31 pediatric hematology-oncology patients with CRBSIs. They suggested that CNS was the most common cause of CRBSIs due to hand contamination during catheter manipulations [19]. In the study by Kar et al., data belonging to 68 children who received chemotherapy for malignancy were analyzed. Although they found that gram-negative bacteria (47.2%) was common, *Staphylococcus* spp. and CNS were the most commonly isolated pathogens in blood culture [20]. In another study conducted in Turkey, Aslan et al. reported that gram-positive cocci (56.4%), gram-negative bacilli (18.9%), and fungi (12.7%) were responsible for BSIs in children with febrile neutropenia. In addition, CNS was the most commonly isolated pathogen [21]. 

Nowadays mortality and morbidity may be reduced with appropriate empirical antimicrobial therapy [22]. However, it is important to identify the antimicrobial susceptibilities of the flora in every center. We found that susceptibility of third generation cephalosporin was 66.7% for *Enterobacter *spp., and this rate was lower in *E. coli*, *Pseudomonas *spp., and *Klebsiella* spp. (42.1%, 50%, and 33.3%, respectively). Susceptibilities of piperacillin-tazobactam and cefoperazone-sulbactam were very high among gram-negative microorganisms; therefore, these agents are used as the first choice in our clinic. 

Extended spectrum beta-lactamase-producing and carbapenem-resistant Enterobacteriaceae may cause difficulty in treatment. In the study by Kapoor et al., ESBL-producing microorganisms such as *E. coli* (4/5), *Klebsiella *spp. (9/10), and *Pseudomonas *spp. (4/4) were isolated from blood culture. However, *E. coli* and *Pseudomonas *spp. did not have any carbapenem resistance and *Klebsiella* spp. had 40% carbapenem resistance [23]. In a study that investigated febrile neutropenia in children with cancer in Turkey, ESBL production was reported as 40.0% and 20.0% for *E. coli *and *Klebsiella *spp., respectively [24]. Thacker et al. detected the following ESBL producing microorganisms: *E. coli* (47.8%), *Acinetobacter* spp. (42.1%), and *Klebsiella *spp. (34.8%). Carbapenem resistance was detected at the rate of 47.8% in *Klebsiella* spp. and 17% in *Pseudomonas* spp. They suggested that carbapenem resistance increased more than ESBL production because of the frequent use of carbapenems in recent years [16]. Our results were better than in this study. We believe that our hospital had lower carbapenemase rates due to avoidance of unnecessary use of carbapenem.

Gram-positive pathogens are an important problem in healthcare-associated infections. Identifying the causative agents may be sometimes difficult due to the isolated pathogen being a part of the normal skin flora [25]. Although different infection rates have been reported in many studies from our country, methicillin-resistant *S. aureus* is frequently isolated in many centers [21]. In a study by Celkan et al., 50% of *S. aureus* had methicillin resistance in BSIs [26]. Vancomycin resistance is another problem in BSIs. In this study, *S. aureus* did not have vancomycin resistance, but resistance was detected in *Enterococcus *spp. at the rate of 33.3%. In the study by Aslan et al., *S. aureus* did not have any vancomycin resistance, but resistance was found in 40% of *Enterococcus *spp. infections, similar to our findings [21]. In our hospital, antibiotic resistance in gram-positive microorganisms was determined to be lower than in other studies. We believe that this is related to the prevention of unnecessary use of glycopeptides.

Multidrug resistance of gram-negative microorganisms can cause very severe infections and poor clinical outcomes in immunocompromised patients [27]. In our study, ceftriaxone susceptibility patterns of *E. coli*,* Klebsiella *spp., and *Pseudomonas* spp. were 42.1%, 33.3%, and 50%, and meropenem susceptibility patterns of these microorganisms were 78.9%, 86.7%, and 50%, respectively. However, all of these microorganisms were found to be sensitive to colistin. 

Kapoor et al. retrospectively evaluated 50 critically ill children who received intravenous colistin treatment. They detected that favorable outcome was 72%, and 14 (28%) children died due to sepsis. Although renal toxicity occurred in five patients, they suggested that in critically ill patients, intravenous colistin is useful for treatment of multidrug resistance gram-negative bacterial infections [28]. In the study by Iosifidis et al., data belonging to 13 patients with 19 episodes in which colistin was used for infections caused by multidrug-resistant nosocomial gram-negative bacteria were analyzed. Favorable outcomes were obtained in 16 (84.2%) patients. Two patients died due to severe sepsis and one patient died because of underlying disease. They found that concomitant use of colistin with other antimicrobial drugs may result in good clinical outcomes in most patients [29]. These results support the findings that the use of empirical colistin should be preferred in critically ill patients with severe clinical signs who do not respond to standard antimicrobial therapy because of the increasing antibiotic resistance patterns.

There are some limitations in our work. The most important limiting factor was that the study was retrospective. In addition, the patients may have been admitted to another center with fever. For this reason, we may have not included the patient in the study. However, we believe that our work will lead to future studies involving a larger number of patients.

In conclusion, BSIs are a serious problem in children during the treatment of the primary disease. *E. coli* and CNS were the most commonly responsible pathogens and beta-lactam/lactamase inhibitors were suitable for empirical treatment. According to antimicrobial susceptibility, sufficient time and dose of intravenous antibiotics were applied, and clinical results were very satisfactory.
